# Induction of Functional Hair-Cell-Like Cells from Mouse Cochlear Multipotent Cells

**DOI:** 10.1155/2016/8197279

**Published:** 2015-12-29

**Authors:** Quanwen Liu, Yi Shen, Jiarong Chen, Jie Ding, Zihua Tang, Cui Zhang, Jianling Chen, Liang Li, Ping Chen, Jinfu Wang

**Affiliations:** ^1^Institute of Cell and Development, College of Life Sciences, Zhejiang University, Hangzhou 310058, China; ^2^Department of Epidemiology & Health Statistics, School of Medicine, Zhejiang University, Hangzhou 310058, China; ^3^Departments of Cell Biology and Otolaryngology, Emory University School of Medicine, Atlanta, GA 30322, USA

## Abstract

In this paper, we developed a two-step-induction method of generating functional hair cells from inner ear multipotent cells. Multipotent cells from the inner ear were established and induced initially into progenitor cells committed to the inner ear cell lineage on the poly-L-lysine substratum. Subsequently, the committed progenitor cells were cultured on the mitotically inactivated chicken utricle stromal cells and induced into hair-cell-like cells containing characteristic stereocilia bundles. The hair-cell-like cells exhibited rapid permeation of FM1-43FX. The whole-cell patch-clamp technique was used to measure the membrane currents of cells differentiated for 7 days on chicken utricle stromal cells and analyze the biophysical properties of the hair-cell-like cells by recording membrane properties of cells. The results suggested that the hair-cell-like cells derived from inner ear multipotent cells were functional following differentiation in an enabling environment.

## 1. Introduction

Cochlear hair cells are terminally differentiated cells that serve as mechanosensory receptors and convert sound stimuli into electric signals [1]. Hair cells in the mammalian inner ear are located in the cochlear organ of Corti and in the vestibular sensory epithelia of the saccular macula, utricular macula, and cristae of the three semicircular canals [2]. These hair cells are susceptible to damage from noise trauma, aging, and aminoglycoside ototoxicity [3]. Loss of hair cells in higher vertebrates appears to be nonreversible and leads to permanent hearing loss [4]. Therefore, restoration of mammalian hearing requires replacement of lost/damaged hair cells either by* in vivo* regeneration or by transplantation of precursor cells capable of implantation and hair cell formation. The generation of new hair cells from a renewable source of progenitors is the principal requirement for development of a cell-based therapy within this sensory organ [5].

Previous reports showed that multipotent cells isolated from the neonatal cochlea as well as adult vestibular sensory epithelia could be differentiated into inner ear hair cells [6, 7]. Therefore, it is likely that inner ear multipotent cells are the suitable source for generating sensory hair cells. However, attempts to obtain equivalent cells from the adult mouse cochlea have not succeeded. The proliferative capacity of cochlear multipotent cells decreases by 100-fold during the second and third postnatal weeks. Therefore, an ideal strategy would utilize early neonatal stages. The neonatal mouse cochlea harbors multipotent cells that retain most of their undifferentiated features if cultured under appropriate conditions [7]. Here, we isolated multipotent cells from the neonatal mouse cochleae. By using defined culture conditions, these multipotent cells showed the ability to form spheres, and the spheres could be passaged [2, 6, 8]. The main goal of our study was to induce the differentiation of inner ear multipotent cells into functional hair cells with stereocilia bundles responsive to voltage stimulation. In most of previous studies, inner ear multipotent cells were induced to differentiate into cells expressing hair cell markers by adhesion on substrates, such as poly-D-lysine, poly-L-lysine, fibronectin, and laminin [1, 7, 9, 10]. In our studies, the similar method was not sufficient to effectively generate functional hair cells with stereocilia bundles. To promote the differentiation potentials of inner ear multipotent cells into functional hair-cell-like cells, we improved the induction method by coculturing inner ear progenitor cells differentiated from mouse cochlear multipotent cells with mitotically inactivated chicken utricle stromal cells. This two-step-induction method promoted the differentiation of inner ear multipotent cells into functional hair cells at a high efficiency. The differentiated cells showed the expression of hair cell markers and the morphology of hair bundles. Furthermore, these hair-cell-like cells were responsive to voltage stimulation and expressed functional mechanotransduction channels [11].

## 2. Materials and Methods

### 2.1. Isolation of Multipotent Cells from the Inner Ear and Sphere Formation

The cochlear sensory epithelia were dissected from postnatal day 0 (P0) ICR mice and incubated in phosphate-buffered saline (PBS) at pH 7.4. The surrounding epithelial tissue and nerve fibers were carefully removed. For preparation of each cell suspension, the sensory epithelia from four cochleae were treated for 7 minutes with 0.05% trypsin (Gibco-BRL, Hangzhou, China) in PBS at 37°C in a total volume of 100 *μ*L. The enzymatic reaction was blocked by adding 100 *μ*L of soybean trypsin inhibitor (1 mg/mL, Sigma), and the lysates were triturated by pipetting up and down 30–40 times with an Eppendorf pipette tip, ensuring that no bubbles were generated. The separation of cells was verified by microscopic inspection. This study was performed in strict accordance with the recommendations in the Guide for the Care and Use of Laboratory Animals of the National Institutes of Health. The protocol was approved by the Committee on the Ethics of Animal Experiments of the medical department of Zhejiang University. All surgeries were performed under sodium pentobarbital anesthesia, and all efforts were made to minimize animal suffering.

Each cell suspension was combined with 2 mL sphere culture medium consisting of DMEM/F12 (mixed 1 : 1) supplemented with N2 (1 : 100) and B27 (1 : 50) (Invitrogen, Shanghai, China), epidermal growth factor (EGF, 20 ng/mL), basic fibroblast growth factor (bFGF, 20 ng/mL), insulin-like growth factor-1 (IGF, 50 ng/mL) (Peprotech, Hangzhou, China), and ampicillin (50 *μ*g/mL). The diluted cell suspension was passed through a 70 *μ*m cell strainer (BD Labware, Shanghai, China) to prepare the suspension of individual cells devoid of large cell aggregates, and 2 mL of the cell suspension was transferred into a 20 *μ*m cell strainer (Haimen, Zhenjiang, China) placed in a fresh well of a six-well suspension plate (Corning, Shanghai, China). This method eliminated differentiated cells such as hair cells and supporting cells that are greater than ~30 *μ*m in size. The cell suspension was cultured in a CO_2_ incubator for 5 days to form the cell spheres.

For cell expansion, we collected individual spheres, mechanically dissociated the cells (and treated briefly with 0.05% trypsin), and replated the cells at a density of 10^5^/mL. Secondary and tertiary sphere formation occurred within 5 days of plating. To determine whether sphere cells were generated by mitosis, thymidine analog 5-bromo-2′-deoxyuridine (BrdU) (Sigma) was added to the media to label the mitotically active cells during sphere formation. Labeling was performed with 5 *μ*M of BrdU throughout the sphere-formation period. Incorporated BrdU was immunochemically detected by labeling with a monoclonal anti-BrdU antibody (Sigma). Quantification was determined by the fraction of BrdU-positive cells among all cells in each sphere.

### 2.2. Preparation of Embryonic Chicken Utricle Stromal Cells and Differentiation of Hair-Cell-Like Cells

Utricles were dissected from embryonic day 18 (E18) chicken and treated with DMEM/F12 (Invitrogen) supplemented with 0.5 mg/mL thermolysin (Sigma) at 37°C for 40 minutes. After adding 5% serum, the sensory epithelia were removed. The remaining pieces of the stromal tissue were transferred into 50 *μ*L of PBS in a six-well culture plate using forceps, combined with 50 *μ*L of prewarmed 0.25% Trypsin/EDTA (Gibco-BRL), and incubated at 37°C for 7 minutes in a CO_2_ incubator. After adding 100 *μ*L DMEM/F12 media supplemented with 10% FBS, the cells were gently triturated and then cultured in a 10 cm culture dish until 80–90% confluency. At the first passage, cells were collected and filtered through a 70 *μ*m strainer (BD Labware) to remove debris. Cells were expanded twice before using as the stromal cells supporting the differentiation of inner ear hair cells. Prior to performing the inner ear hair cell differentiation experiments, the chicken utricle stromal cells were plated onto gelatin-coated six-well dishes (Corning) and grown until 90% confluency. The cells were then mitotically inactivated with 2 *μ*g/mL mitomycin C in DMEM/F12 with 10% FBS for 3 hours and washed three times with media before use for inner ear hair cell differentiation cultures.

The two-step-induction method was used for inducing hair-cell-like cell differentiation from inner ear multipotent cells. Firstly, the cochlear multipotent cells from the tertiary spheres were plated in 24-well dishes coated with poly-L-lysine and then cultured for 7 days to induce the differentiation of progenitor cells of the inner ear cell lineage. The differentiation medium for the inner ear progenitor cell lineage consisted of DMEM/F12 (Invitrogen, mixed 1 : 1), N2 (1 : 100), B27 (1 : 50) (Invitrogen), FGF3 (50 ng/mL, Invitrogen), and FGF10 (50 ng/mL, Invitrogen). After 7 days of differentiation, most of inner ear multipotent cells have differentiated into progenitor cells. Subsequently, we used two substrates, poly-L-lysine and inactivated embryonic chicken utricle stromal cells, for differentiation of progenitor cells into hair cells. For the differentiation on poly-L-lysine, the progenitor cells were continued to be cultured in 24-well dishes coated with poly-L-lysine for another 7 days. For the differentiation on inactivated embryonic chicken utricle stromal cells, the inner ear progenitor cells were separated by a 7-minute treatment with Accutase (Invitrogen) at 37°C and then cultured in 24-well dishes coated with mitotically inactivated E18 chicken utricle stromal cells for 7 days. The differentiation medium for hair cell differentiation was DMEM/F12 (Invitrogen, mixed 1 : 1) medium supplement with N2 (1 : 100), B27 (1 : 50), and DAPT (5 *μ*M, Sigma). The exhausted medium was replaced every second day. Differentiated cells were analyzed by a combination of total RNA preparation followed by reverse transcription-polymerase chain reaction (RT-PCR), immunocytochemistry, and scanning electron microscopy (SEM) or electrophysiology.

### 2.3. Analysis of Gene Expression Specific for Inner Ear Multipotent Cells and Inner Ear Hair Cells

Cells from tertiary spheres and cells differentiated for 7 and 14 days were analyzed for expression of specific genes using RT-PCR. Total RNA was extracted using Trizol reagent (TaKaRa, Shanghai, China) according to manufacturer's instructions. RNA (1 *μ*g) was used to synthesize first-strand cDNA by reverse transcriptase (Fermentas, Shanghai, China), and the expression levels of the Nestin, Abcg2, Pax-2, BMP-4, BMP-7, Myosin VIIA, Espin, Brn3c, and P27^kip1^ genes were determined. GAPDH was used as a housekeeper gene for normalization of all measurements. Individual primers across two or more exons were designed using the Primer Express 1.0a software (Applied Biosystems) to discriminate between cDNA and genomic DNA. The resulting cDNAs were used as templates for PCRs using the following primer pairs (forward, reverse, cDNA product length): GAPDH (5′-AAC GGG AAG CCC ATC ACC-3′, 5′-CAG CCT TGG CAG CAC CAG-3′, 442 bp), Nestin (5′-GGA GAG TCG CTT AGA GGT GC-3′, 5′-CTT GGG GTC AGG AAA GCC AA-3′, 334 bp), Abcg2 (5′-GCT GTG GAG CTG TTC GTA GTG G-3′, 5′-GCT AAA GTA CTG AAG CCA GGA C-3′, 387 bp), Pax-2 (5′-CCA AAG TGG TGG ACA AGA TTG CC-3′, 5′-GGA TAG GAA GGA CGC TCA AAG AC-3′, 475 bp), BMP-4 (5′-TGG TAA CCG AAT GCT GAT GGT CG-3′,5′-GTC CAG TAG TCG TGT GAT GAG GTG-3′, 598 bp), BMP-7 (5′-TGG GCT TCT GAG GAG GGC TGG TTG-3′,5′-TGG CGT GGT TGG TGG CGT TCA T-3′, 484 bp), Myosin VIIA (5′-CTC CCT CTA CAT CGC TCT GTT CG-3′, 5′-AAG CAC CTG CTC CTG CTC GTC CAC G-3′, 628 bp), Espin (5′-CAG CCT GAG TCA CCG CAG CCT C-3′, 5′-TGA CCT GTC GCT GCC AGG GCG CG-3′, 475 bp), Brn3c (5′-GCC ATG CGC CGA GTT TGT C-3′, 5′-ATG GCG CCT AGA TGA TGC-3′, 368 bp), P27^kip1^ (5′-CTG GAG CGG ATG GAC GCC AGA C-3′, 5′-CGT CTG CTC CAC AGT GCC AGC-3′, 525 bp). The PCR conditions were 94°C for 5 min, then 35 cycles of 94°C for 30 s, 58°C (Nestin), 58°C (Abcg2), 60°C (Pax-2), 60°C (BMP-4), 60°C (BMP-7), 62°C (Myosin VIIA), 62°C (Espin), 60°C (Brn3c), 62°C (P27^kip1^), and 58°C (GAPDH) for 30 s, and then 72°C for 30 seconds, finally followed by 72°C for 7 minutes. PCR products were electrophoresed in a 1.2% agarose gel and then stained with ethidium bromide. Each PCR product was resolved three times. Stained bands were visualized under UV light and photographed. Band intensities were analyzed quantitatively in triplicates using the ImageJ software, and the expression level of each gene was normalized to that of GAPDH.

### 2.4. Immunolabeling

For immunolabeling, cells were fixed in prechilled PBS with 4% paraformaldehyde for 15 minutes and permeabilized in PBS with 0.25% Triton X-100 for 10 minutes at room temperature. Nonspecific binding sites were blocked for 1 hour by PBS with 1% bovine serum albumin and 0.1% Tween 20. The fixed cells were incubated overnight at 4°C with antibodies specific for BrdU (1 : 200, rabbit polyclonal, Sigma); Nestin (1 : 200, rabbit polyclonal, Abcam, Hangzhou, China); Pax-2 (1 : 100, rabbit polyclonal, Abcam); Pax-8 (1 : 10, mouse monoclonal, Abcam); Atoh1 (1 : 300, rabbit polyclonal, Abcam); Myosin VIIA (1 : 200, rabbit polyclonal, Abcam); Espin (1 : 200, rabbit polyclonal, Santa Cruz, Hangzhou, China); Brn3c (1 : 32, mouse monoclonal, Abcam); P27^kip1^ (1 : 1600, mouse monoclonal, Cell Signaling, Hangzhou, China); *α*-tubulin (1 : 200, mouse monoclonal, Invitrogen); and FITC-conjugated phalloidin complex (50 *μ*g/mL, Sigma). Specific labeling was visualized using secondary donkey anti-mouse, anti-goat, or anti-rabbit antibodies conjugated to either Alexa Fluor 488 or Alexa Fluor 568 (Jackson, Hangzhou, China). Nuclei were visualized by staining with 4′,6-diamidino-2-phenylindole (DAPI).

Hair cells exhibit rapid permeation to FM1-43FX, a dye that passes through open mechanotransduction channels [12]. To test the possibility that the differentiated cells expressed functional mechanotransduction channels, cells differentiated for 14 days were incubated in DMEM-F12 containing FM1-43FX (5 *μ*M; Invitrogen) for 10 seconds at room temperature, washed thoroughly in fresh DMEM/F12 medium, fixed in 4% paraformaldehyde, counterstained with DAPI, and finally observed under the fluorescence microscope. Furthermore, immunostaining for Myosin VIIA was performed following FM1-43FX dye treatment to confirm the identity of hair cells.

### 2.5. SEM

Cells differentiated for 14 days were fixed overnight at 4°C with 0.1 M phosphate buffer with 2.5% glutaraldehyde (Sigma). After fixation, the specimens were rinsed with PBS followed by postfixation for 30 minutes with 1% osmium tetroxide (Sigma). The specimens were washed three times between each treatment step and then dehydrated in a graded ethanol series. Thereafter, the ethanol was replaced by isoamyl acetate (Aladdin, Shanghai, China) for 20 minutes and dried by critical point drying. Specimens were viewed with a Hitachi S-3000N variable pressure SEM operated under high vacuum at 5–10 kV at a working distance of 7–10 mm.

### 2.6. Electrophysiological Recordings

The whole-cell patch-clamp technique was used to measure the membrane currents of cells differentiated for 14 days using the Multiclamp 700B amplifier (Molecular Devices, Union City, CA). Recordings were obtained at 30°C using solution inline heater (Warner Instruments). The extracellular solution contained (mM) 135 NaCl, 5.8 KCl, 1.3 CaCl_2_, 0.9 MgCl_2_, 0.7 NaH_2_PO_4_, 5.6 D-glucose, 10 HEPES-NaOH, and 2 sodium pyruvate. The pH was adjusted to 7.5. Cells were viewed using an upright microscope and continuously superfused with extracellular solution. Patch pipettes were pulled from soda glass capillaries (3-4 MΩ) and coated with surf wax. The pipette filling solution contained (mM) 131 KCl, 3 MgCl_2_, 1 EGTA-KOH, 5 Na_2_ATP, 5 HEPES-KOH, and 10 sodium phosphocreatine (pH 7.3).

Cells differentiated for 14 days were incubated in DMEM-F12 containing FM1-43FX for 10 seconds, and the cells labeled with FM1-43FX showed green fluorescence. Only cells with green fluorescence were used for electrophysiological recording. Positive pressure was maintained as the recording pipette was lowered into the epithelium. When the recording pipette touched the membrane, positive pressure was released, forming a tight seal. Membrane currents were elicited by applying voltage steps in 10 mV nominal increments or decrements from the holding potential of −84 mV and stepped between −120 and 50 mV. The data were acquired using the pClamp software and a Digidata analog-to-digital converter (Molecular Devices). Data were filtered at 2.5 or 5 kHz, sampled at 5 or 50 kHz, and stored on a computer for offline analysis using the Clampfit and Origin (OriginLab) software.

### 2.7. Statistical Analysis

Results were expressed as means ± standard deviation (SD) of the means of samples. All collected data were examined by multifactorial analysis of variance. Differences between the independent variables were assessed in post hoc tests (Tukey's studentized range tests for variables). All tests were two-tailed and statistical significance was set at *P* < 0.05.

## 3. Results

### 3.1. Establishment of Multipotent Cell Spheres from the Neonatal Mouse Cochlear Epithelia

**Figure 1:**
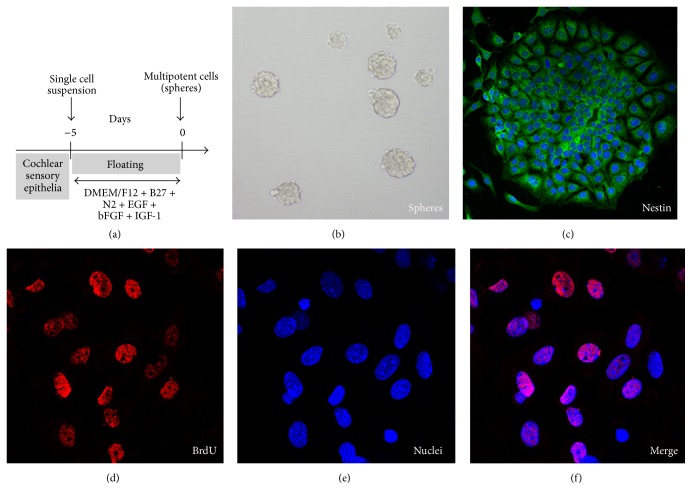
Characterization of spheres from the cochlear epithelia of newborn mice. (a) The cochlear sensory epithelia of P0 ICR mice were dissociated into single cells and cultured in suspension plates for 5 days to form spheres in presence of EGF, bFGF, and IGF-1, as indicated. (b) A bright-field view of a typical sphere generated after 5 days of* in vitro* culture. (c) Sphere cells expressed the neuronal stem cell marker Nestin (green). (d) Shortly after dissociation, most sphere cells were immunoreactive for 5-bromo-2′-deoxyuridine (BrdU) incorporation into the genomic DNA (red, left). (e) The nuclei of the sphere cells were stained with DAPI (blue). (f) The merging of BrdU immunostaining (red) and DAPI staining (blue).

The cochlear sensory epithelia were dissected from P0 ICR mice, and cells isolated from the sensory epithelia prepared the cell suspension (Figure 1(a)). This procedure generally yielded a completely dissociated individual cell suspension devoid of aggregates and eliminated differentiated cells such as hair cells and supporting cells. Finally, an aliquot of cell suspension was transferred into a plastic culture dish under adherent conditions and immunolabeled with Myosin VIIA antibody to determine whether hair cells remained in the cell suspension. The results showed that no Myosin VIIA-positive cells were detected.

After the cells from cochlear epithelia were cultured for 5 days, the spheres were formed with a frequency of 250 ± 50 spheres per 10^5^ cells plated (mean ± SD, *n* = 4; Figure 1(b)). We observed that cells derived from cochlear epithelia initially give rise to small solid spheres, subsequently become transitional spheres, and finally form hollow spheres. We found that 5-day-old solid spheres harbored 85 ± 5 cells (mean ± SD, *n* = 5), whereas the number of cells in 5-day-old hollow spheres was only 43 ± 6 (mean ± SD, *n* = 5). To determine whether sphere cells were generated by mitosis, we used the thymidine analog BrdU to label mitotically active cells during sphere formation. The results showed that 85.3 ± 5.2% of sphere cells had replicated DNA and their nuclei were labeled with BrdU (mean ± SD, *n* = 5; Figures 1(d), 1(e), and 1(f)). These observations indicated that neonatal cochlear epithelia contained cells with the potential to proliferate and form spheres.

To determine whether the sphere-forming cells from cochlear epithelia are capable of proliferation, we dissociated the spheres and cultured the individual cells at a low density. Secondary spheres were formed at a frequency of 2.0 ± 0.5 spheres (*n* = 4) from cells derived from a single primary sphere. Finally, tertiary spheres formed at a frequency of 1.5 ± 0.5 (*n* = 4). The tertiary spheres were not expandable by additional passages, likely due to their limited proliferation capability.

Nestin is a class IV intermediate filament expressed throughout nervous system development and is commonly associated with neural stem cells [13]. Therefore, it has been used to characterize stem cells isolated from early postnatal cochlea [14]. To determine whether the tertiary spheres derived from cochlear epithelia contained stem cells, whole spheres were immunostained to detect Nestin. We found that the majority of sphere cells expressed Nestin (Figure 1(c)), indicating that spheres formed from mouse cochlear sensory epithelia cells consisted of, at the least, certain features of stem cells.

**Figure 2:**
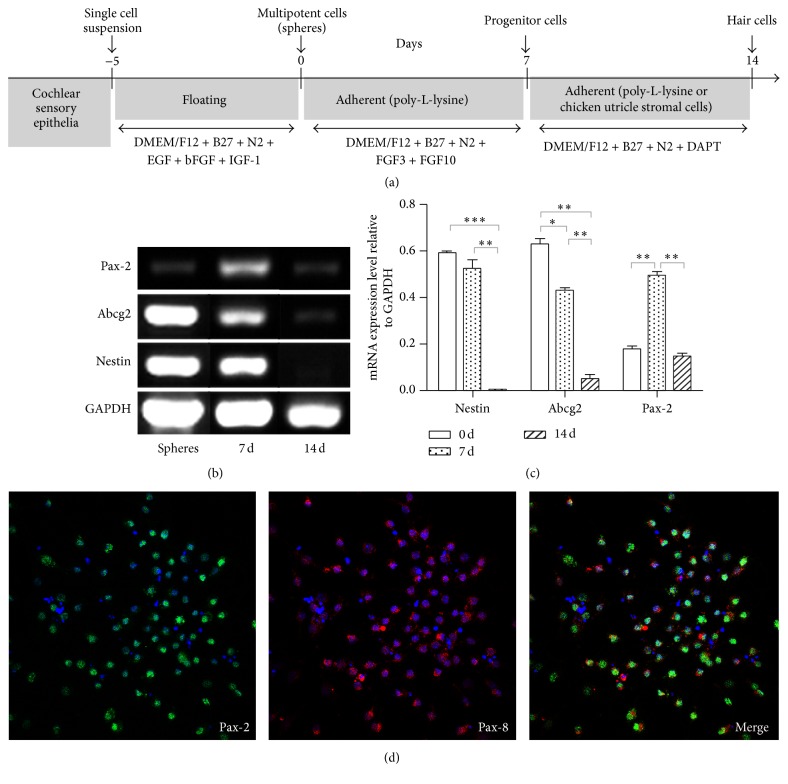
Gene expression of sphere cells and cells differentiated for 7 and 14 days on poly-L-lysine. (a) Spheres were generated for 5 days in presence of EGF, bFGF, and IGF-1, as indicated. Progenitor cells were generated by subsequent adherent culture for 7 days in poly-L-lysine coated plates in presence of FGF3 and FGF10. On day 7, the resulting progenitor cells were grown adherently on a poly(L-lysine) substratum or a layer of mitotically inactivated chicken utricle stromal cells for 7 days to form hair cells in presence of DAPT. (b) Reverse transcription-polymerase chain reaction (RT-PCR) expression analysis of markers of stem cells and progenitor cells of inner ear cell lineages. The ubiquitously expressed GAPDH is shown as a reference. (c) Expression of the stem cell markers Nestin and Abcg2 and the inner ear progenitor cell marker Pax-2. Spheres: spheres generated from cochlear multipotent cells, 7 d: cells differentiated for 7 days on the poly-L-lysine substratum* in vitro*, and 14 d: cells differentiated for 14 days on the poly-L-lysine substratum* in vitro*. Statistically significant differences between groups are indicated by ^*∗*^ *P* < 0.05, ^*∗∗*^ *P* < 0.01, and ^*∗∗∗*^ *P* < 0.001 (*n* = 3). (d) Coexpression of the inner ear progenitor cell markers, Pax-2 (red, left) and Pax-8 (green, center), in the sphere cells. Right: merged images (left and center).

To assess the differentiation of cells toward the inner ear precursor cell lineage from the spheres (Figure 2(a)), in addition to Nestin immunostaining, we analyzed the expression of Nestin, another stem-cell marker Abcg2, and inner ear progenitor-cell marker gene Pax-2 [15] in tertiary spheres and differentiating precursor cell cultures by using semiquantitative RT-PCR (Figures 2(b) and 2(c)). The Nestin mRNA levels in cells from tertiary spheres and 7-day culture groups (inner ear multipotent cells differentiated for 7 days on the poly-L-lysine substratum) were significantly higher than those in the 14-day culture group (inner ear multipotent cells differentiated for 14 days on the poly-L-lysine substratum) (*P* < 0.05). However, no significant difference in Nestin expression was detected between the sphere group and the 7-day culture group. The expression level of Abcg2 in cells from the sphere group was significantly higher than that in the 7-day culture group (*P* < 0.05) and the 14-day culture group (*P* < 0.01). Therefore, the expressions of stem cell marker genes Abcg2 and Nestin were significantly downregulated in the cells differentiated for 2 weeks. The expression level of Pax-2 in cells from the 7-day culture group was significantly higher than that in spheres and 14-day culture group (*P* < 0.01). The results showed that some multipotent cells in the spheres had developed into the progenitor cell stage after 7 days of differentiation. After 14 days of differentiation, some progenitor cells in the 7-day culture group differentiated into mature cells, such as hair cells and supporting cells, reflected by the downregulation of Pax-2 expression in the 14-day culture group. The expression level of Pax-2 showed no significant differences between sphere group and 14-day culture group. In addition, the expression levels of BMP-4 and BMP-7 showed no significant differences among the three groups (data not shown).

### 3.2. Hair Cell Differentiation Induced on Poly-L-Lysine Substratum

**Figure 3:**
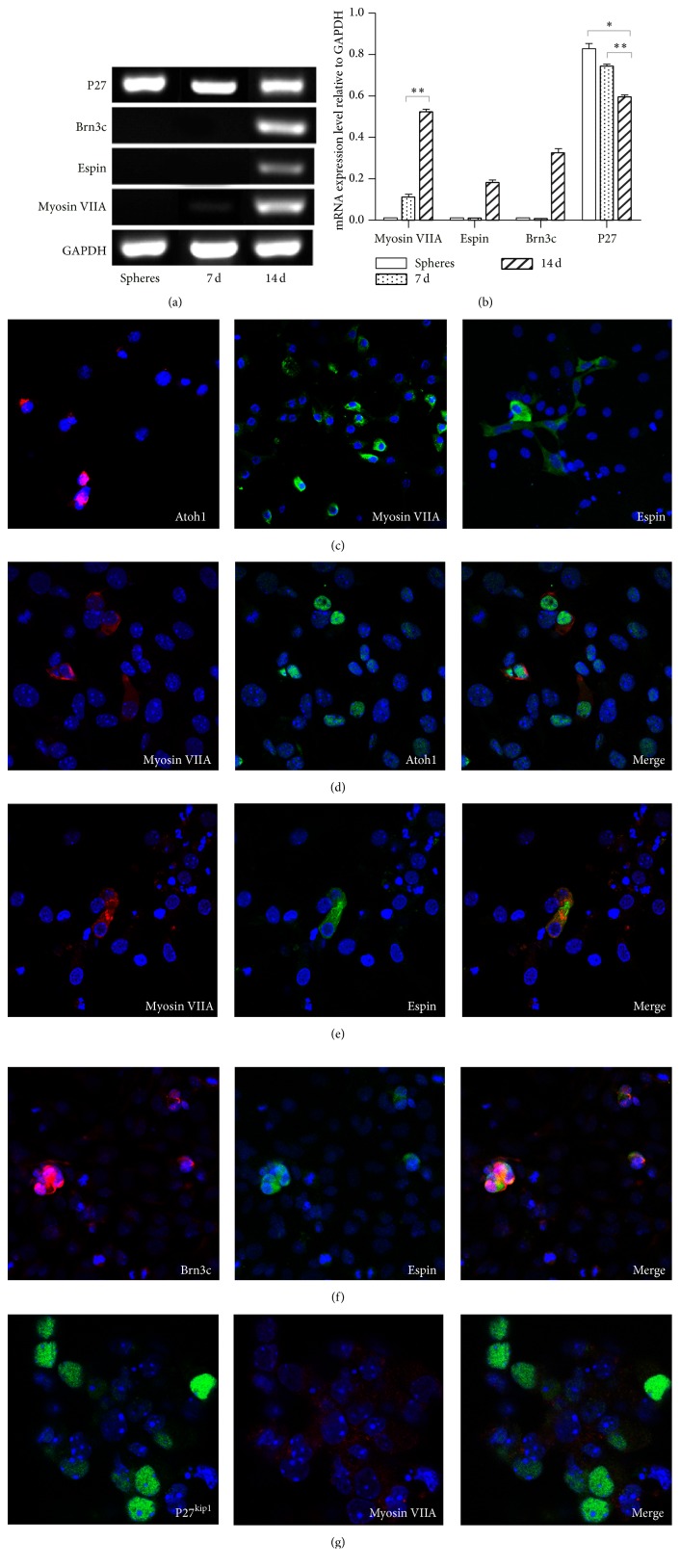
Molecular characterization of inner ear multipotent cells and derived cells differentiating on poly-L-lysine substratum. (a) RT-PCR analysis of the expression of markers specific for hair cells and supporting cells. The ubiquitously expressed GAPDH is shown as reference. (b) Expression of markers specific for hair cells and supporting cells: Myosin VIIA, Brn3c, Espin, and P27^kip1^. Spheres: spheres generated from inner ear multipotent cells, 7 d: cells differentiated for 7 days on the poly-L-lysine substratum* in vitro*, and 14 d: cells differentiated for 14 days on the poly-L-lysine substratum* in vitro* (as shown in Figure 2(a)). Statistically significant differences between groups are indicated by ^*∗*^ *P* < 0.05, ^*∗∗*^ *P* < 0.01, and ^*∗∗∗*^ *P* < 0.001 (*n* = 3). (c) After 5 days of differentiation, Atoh1-positive cells appeared in differentiating sphere-derived cells (red, left). After 2 weeks of differentiation (as shown in Figure 2(a)), hair-cell-like cells appeared in cells derived from spheres. 25 ± 3.1% (*n* = 5) of differentiated cells were labeled with an antibody to Myosin VIIA (green, center), and 18 ± 3.7% (*n* = 5) of differentiated cells were labeled with an antibody to Espin (green, right). Nuclei were visualized with DAPI. (d) All cells immunopositive for Myosin VIIA also expressed Atoh1. (e) Myosin VIIA-positive cells in stem-cell-derived cell populations coexpressed the stereocilia marker Espin. (f) The Espin-positive cells in stem-cell-derived cell populations coexpressed Brn3c. (g) Conventional fluorescence microscopy showing nonoverlapping expression of p27^kip1^ (green) and Myosin VIIA (red) in differentiated cells.

The expression of markers for inner ear early development led us to explore whether sphere cells could give rise to hair cells and supporting cells. Adherent and serum-free cultures provide an effective means of initiating differentiation from multipotent cells. In this study, tertiary spheres were incubated on a poly(L-lysine) substratum for 2 weeks by the two-step-induction method (Figure 2(a)). The relative expression levels of markers specific for hair cells were examined by RT-PCR (Figure 3). No expression of mature hair cell markers (Myosin VIIA, Brn3.1, and Espin) was detectable in the tertiary spheres derived from cochlear epithelia (Figures 3(a) and 3(b)). After 7 days of differentiation, the expression of Pax-2 mRNA was upregulated (Figures 2(b) and 2(c)). Meanwhile, the immunolabeling assay with antibodies specific for the inner ear progenitor cell markers Pax-2 and Pax-8 showed that 81.5 ± 3.6% of sphere cell-derived cells were positive for expression of Pax-2 and Pax-8 (mean ± SD, *n* = 5; Figure 2(d)). It indicated that most cells have differentiated into cells with characteristics of the inner ear progenitor cell lineage. Interestingly, the cells differentiated for 7 days showed initiation of Myosin VIIA expression (Figures 3(a) and 3(b)). After 2 weeks of differentiation, the expression of Myosin VIIA mRNA was upregulated, and Brn3c and Espin mRNAs were expressed (Figures 3(a) and 3(b)). Notably, the expression of marker specific for the progenitor cells, such as Pax-2, was sustained at a lower level (Figures 2(b) and 2(c)), implying that some cells may remain as precursor cells.

The expression of P27^kip1^ is expressed in the progenitors of both supporting cells and hair cells and subsequently sustained in supporting cells but downregulated in differentiating hair cells. To examine the development of supporting cells, the expression level of P27^kip1^ was detected using semiquantitative RT-PCR. The results showed that P27^kip1^ transcript was present at a low level in the sphere group and upregulated in the 7-day culture group. P27^kip1^ expression was downregulated in the 14-day culture group (Figures 3(a) and 3(b)). After 7 days of differentiation, the number of progenitors increased, resulting in the upregulation of P27^kip1^ expression. Meanwhile, some progenitors may have differentiated into hair-cell-like cells, reflected by the presentation of Myosin VIIA expression in the 7-day culture group. After 14 days of differentiation, hair-cell-like cells increased, resulting in upregulation of Myosin VIIA expression and downregulation of P27^kip1^ expression.

Due to the inherent heterogeneity of multipotent cell spheres and subsequent cultures, RT-PCR is suitable only to a limited extent for the analysis of a trend change among different cultures. We further used antibodies to examine the expression of Atoh1, a basic helix-loop-helix transcription factor, and a homolog of the* Drosophila atonal* gene, a key transcription factor for hair cell differentiation [16]. No Atoh1-positive cells were detected in the tertiary spheres. Atoh1-expressing cells were detected after 5 days of* in vitro* differentiation (Figure 3(c), red, left), and the number of Atoh1-expressing cells was increased progressively in the differentiation cultures. The number of cells expressing more mature hair cell markers was also increased in longer cultures. 25.2 ± 3.1% and 18.6 ± 3.7% of cells differentiated for 14 days on poly-L-lysine were expressed in Myosin VIIA (mean ± SD, *n* = 5; Figure 3(c), green, center) and Espin (mean ± SD, *n* = 5; Figure 3(c), green, right), respectively. Furthermore, we found that Atoh1 was expressed only in cells exiting the cell cycle, while all Myosin VIIA-positive cells coexpressed Atoh1 (Figure 3(d)) and Espin (Figure 3(e)), which is abundantly expressed in the stereocilia [17]. Brn3c is a transcription factor required for hair cell survival and maturation, but not fate determination and proliferation of hair cells [18]. Our results showed that the Espin-positive cells coexpressed Brn3c that was absent from some Myosin VIIA-positive cells (Figure 3(f)). The overlapping expression of hair cell markers of different developmental stages indicated that hair cell differentiation in the 14-day culture group was heterogeneous. It is worth noting that the efficiency of the formation of Myosin VIIA-positive cells (25.2 ± 3.1%) using the current condition is much higher than that reported previously (8.7 ± 3.8% or 3.6 ± 1.4%) [6, 7].

P27^kip1^ is a protein that is initially expressed in the progenitor cells for both hair and supporting cells and subsequently downregulated in hair cells and retained in the mature supporting cells [19, 20]. In our experiments, P27^kip1^ was detectable only in some cells with no expression of hair cell markers after 14 days of differentiation (Figure 3(g)), suggesting that some multipotent cells should differentiate into supporting cells. Therefore, we performed a control experiment to test whether the supporting cells are reduced in the presence of DAPT. For the progenitor cells grown adherently on a poly(L-lysine) substratum for 7 days without DAPT, there were 30 ± 3.5% of the differentiated cells expressing P27^kip1^. In contrast, only 12.5 ± 2.8% of the differentiated cells were expressed in P27^kip1^ under DAPT treatment. It indicated that DAPT treatment resulted in the reduction of supporting cells.

The appearance of Espin-positive cells suggested that these cells differentiated on poly-L-lysine had developed stereocilia. Therefore, the cell clusters containing Espin-positive cells were analyzed by SEM. No stereocilia were found on the surface of sphere cells that were plated onto poly-L-lysine-treated coverslips by SEM (Figure 5(a)). After 2 weeks of differentiation, cells differentiated on the poly-L-lysine-treated coverslip showed an abundance of stereocilia on the surface of the hair-cell-like cells, albeit the stereocilia were disorganized and did not show a bundle morphology as mature hair cells (Figures 5(b) and 5(c)). This result suggested that these hair-cell-like cells developed certain molecular and characteristics of hair cells but lacked induction guidance for the formation of an organized hair bundle. Furthermore, these Myosin VIIA-positive and Espin-positive cells could not be rapidly permeated by FM1-43FX, indicating that these hair cell-like cells were not functional hair cells.

### 3.3. Induction of Functional Hair Cells from Progenitor Cells Derived from Inner Ear Multipotent Cells

**Figure 4:**
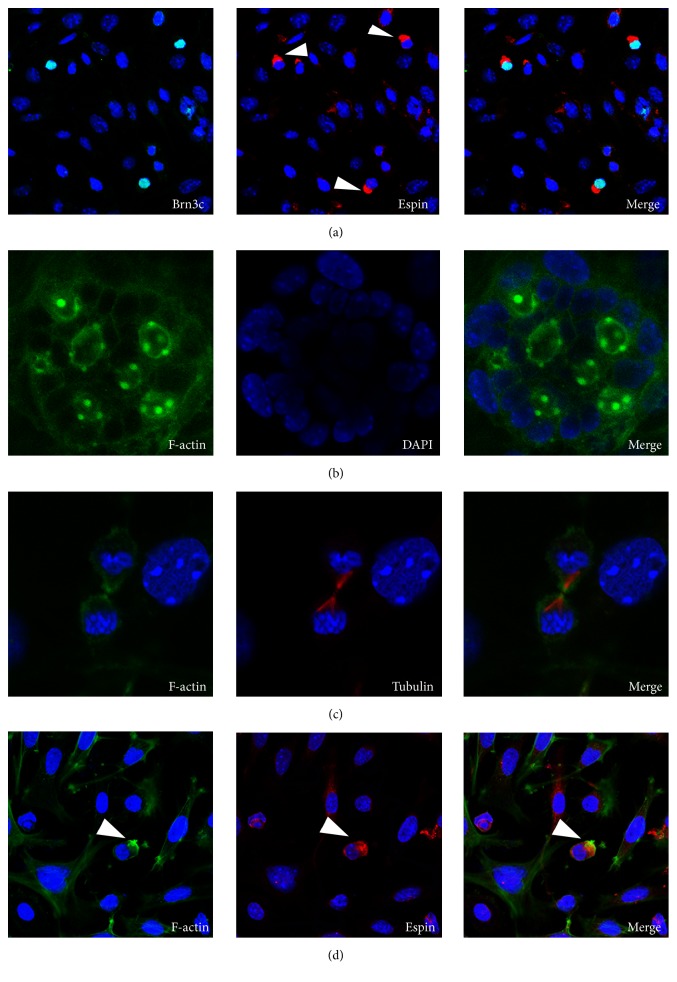
Hair bundle-like protrusions on cells differentiated on chicken utricle stromal cells. (a) The progenitor cells were plated on a layer of mitotically inactivated chicken utricle stromal cells (as shown in Figure 2(a)). After 7 days of differentiation, the Brn3c-positive cells (green) coexpressed the stereocilia marker Espin (red). Espin immunoreactivity was localized on one side of the presumptive hair cells (arrowheads in (a)). (b) When the progenitor cells were cultured on chicken stromal cells (as shown in Figure 2(a)), some cells displayed F-actin-rich protrusions. (c) Bundle-like structures protruding from the surface of the hair-cell-like cells showed colocalization of F-actin (green, left) and tubulin (red, center). Right: merged image of both labels. (d) The stereocilia-like extension was labeled with F-actin (arrowheads in (d), green, left) and Espin (arrowheads in (d), red, center). Right: merged image of both labels.

Cells isolated and expanded from embryonic chicken utricle have been used to generate hair-cell-like cells derived from mouse embryonic stem cells and induce pluripotent stem cells [21, 22]. In order to generate functional hair cells with a characteristic hair cell bundle, we modified the induction method described in previous studies [6, 10, 23] and used cells from embryonic chicken utricles to support the differentiation of inner ear progenitor cells into functional hair cells. Inner ear multipotent cells were induced into progenitor cells in dishes coated with poly-L-lysine, and these progenitor cells were transferred onto a layer of mitotically inactivated chicken utricle stromal cells and induced to differentiate functional hair cells (Figure 2(a)). As a control, the inactivated chicken utricle stromal cells alone cultured for 2 weeks showed no hair cell morphology or expression of hair cell markers (data not shown). However, we observed a different cellular behavior in hair cell markers-positive cells in stromal cell-fed cultures from that in L-lysine-coated 14-day cultures. Brn3c-positive cells harbored in the population of the differentiated cells coexpressed the stereocilia marker Espin (Figure 4(a), green, left), and Espin immunoreactivity showed an asymmetrical distribution of the protein toward one side of the presumptive hair cells (Figure 4(a), red, center). Stereocilia cores consist of F-actin crosslinked by Espin, whereas the kinocilium is tubulin-filled and positioned at the edge of the stereocilia bundle [21]. We observed F-actin labeled by phalloidin in stereocilia-like bundles protruding from cellular clusters cultured on the stromal cells (Figure 4(b)). Using an anti-tubulin antibody to visualize the kinocilia, we found that the kinocilium was located at the edge of each individual hair bundle (Figure 4(c)). We also found that the stereocilia-like extensions were labeled with F-actin and antibody to Espin (Figure 4(d)).

**Figure 5:**
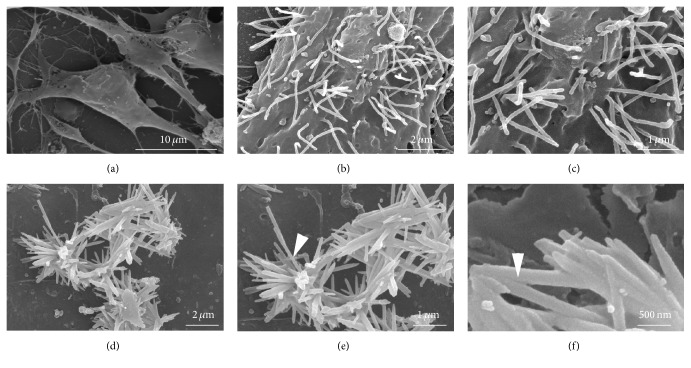
The surface of differentiated cells observed by scanning electron microscopy. (a) The surface of the cochlear multipotent cells had no stereocilia. ((b) and (c)) Cells differentiated on poly-L-lysine (as shown in Figure 2(a)) with some hair-cell-like cells displaying disorganized stereocilia. (d) Cells differentiated on the mitotically inactivated chicken utricle stromal cells (as shown in Figure 2(a)) showed hair bundle-like structures. (e) The stereocilia of the hair-cell-like cells were tapered at their bases (arrowhead in (e)). (f) Many interstereocilia links existed among the tips of stereocilia (arrowhead in (f)).

SEM was used to visualize the morphology of hair bundles on the hair-cell-like cells that were differentiated on mitotically inactivated chicken utricle stromal cells. The hair bundle-like structures on the cell surface showed a regular arrangement (Figure 5(d)). Importantly, the stereocilia of the hair-cell-like cells were tapered at their bases, a hallmark of hair cell stereocilia (Figure 5(e)). In addition, many interstereocilia links as well as links between the tips of stereocilia were observed (Figure 5(f)). These results indicated that coculture of inner ear progenitor cells with mitotically inactivated chicken utricle stromal cells promoted the development of hair bundle-like structures.

**Figure 6:**
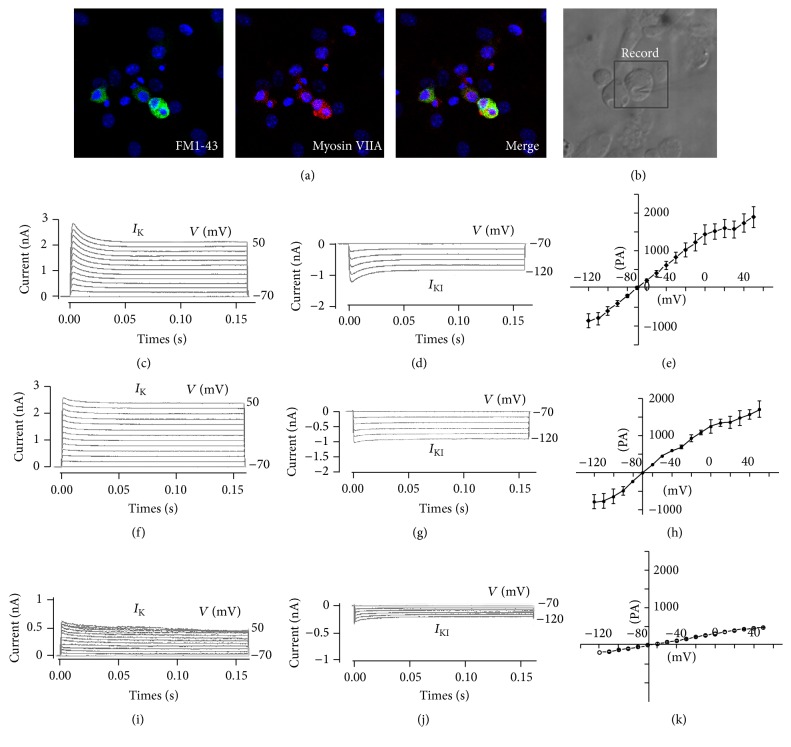
Voltage-dependent currents elicited from inner ear multipotent-cell-derived FM1-43FX-positive cells. FM1-43FX-positive cells were voltage clamped at −84 mV and stepped between −120 and 50 mV in 10 mV increments. 10 out of 18 cells tested had positive responses and expressed the similar outward and inward K^+^ currents. (a) FM1-43FX-positive cells (green, left) coexpressed the hair cell marker Myosin VIIA (red, center). (b) White light image of a recording electrode on an FM1-43FX-positive cell. (c) Outward K^+^ currents recorded from hair-cell-like cells. (d) Inward K^+^ currents recorded from hair-cell-like cells. (e) Average current-voltage (*I-V*) curve for the outward and inward K^+^ current measured of hair-cell-like cells at the steady-state level (100–160 ms). (f) Outward K^+^ currents recorded from neonatal mouse cochlea hair cells. (g) Inward K^+^ currents recorded from neonatal mouse cochlea hair cells. (h) Average current-voltage (*I-V*) curve for the outward and inward K^+^ current of neonatal mouse cochlea hair cells measured at the steady-state level (100–160 ms). (i) Outward K^+^ currents recorded from the cells that are not hair cells. (j) Inward K^+^ currents recorded from the cells that are not hair cells. (k) Average current-voltage (*I-V*) curve for the outward and inward K^+^ current of the cells that are not hair cells measured at the steady-state level (100–160 ms).

To determine whether hair-cell-like cells expressed functional mechanotransduction channels, we incubated the cell clusters differentiated from inner ear progenitor cells on inactivated chicken utricle stromal cells with FM1-43FX. FM1-43FX dye can be taken by the mechanotransduction channels located in the stereocilia and can be used to visualize hair cells with actin-rich hair bundles consisting of mechanotransduction channels [22, 24]. Cellular clusters were incubated for 10 seconds with 5 *μ*M FM1-43FX 3 followed by 10 minutes of rinsing with DMEM/F12. The results showed that many cells had taken up FM1-43FX and nearly all FM1-43FX-labeled cells expressed hair cell marker Myosin VIIA (Figure 6(a)), indicating the emergence of hair cell-like cells with potentially functional mechanotransduction channels. 83 ± 3.6% (mean ± SD, *n* = 5) of the Myosin VIIA-positive cells were labeled with FM1-43FX.

Moreover, we examined whether the hair-cell-like cells with observed hair bundle-like tapped structures connected by interstereocilia links were responsive to voltage stimulation. The FM1-43FX-positive cells were used for recording membrane properties by analyzing voltage-dependent currents (Figure 6(b)). The FM1-43FX-positive cells were voltage clamped at −84 mV and stepped between −120 mV and 50 mV in 10 mV increments. The average current-voltage (*I-V*) curve for the outward (*I*_K_: 1.4 ± 0.2 nA at 0 mV, *n* = 10) and inward (*I*_K1_: −1.8 ± 0.2 nA at −120 mV, *n* = 10) K^+^ current was measured at the steady-state level (100–160 ms). In our experiment, a total of 18 FM1-43-positive cells were successfully recorded and 10 of these cells expressed an outward (Figure 6(c)) and inward (Figure 6(d)) K^+^ current in the presence of K^+^ in the internal solution. Average current-voltage (*I-V*) curve for the outward and inward K^+^ current measured of hair-cell-like cells at the steady-state level (100–160 ms) was shown in Figure 6(e). We isolated the cochlear from neonatal mouse and labeled the hair cells with FM1-43. These hair cells also expressed an outward (Figure 6(f)) and inward (Figure 6(g)) K^+^ current. The slow decay and voltage activation range (Figure 6(e)) of the induced hair cell-like cell resembled recordings in mouse cochlear hair cells (Figure 6(h)). We also detected the electrophysiological profile of the cells that are not HCs. These cells were not responsive to voltage stimulation and had not expressed an outward (Figure 6(i)) and inward (Figure 6(j)) K^+^ current. The average* I-V* curve for the outward and inward K^+^ current measured of these cells at the steady-state level (100–160 ms) was shown in Figure 6(k).

The morphology of hair bundles, the capability to take up FM1-43FX, and the electrophysiological properties of the hair cells differentiated on the mitotic inactive chicken utricle stromal cells suggest that the culture conditions used promote the formation of functional hair cells.

## 4. Discussion

In this study, we developed a modified protocol that allows the effective differentiation of functional hair cells from multipotent cells derived from the cochlear epithelia. The study addresses several key elements in hair cell differentiation* in vitro* from multipotent cells.

### 4.1. Establishment of Proliferating Multipotent Cells from Mouse Cochlear Epithelia

The* in vitro* proliferation is pivotal for obtaining a sufficient population of multipotent cells. Wang et al. [2] found that EGF and bFGF could promote the proliferation of inner ear stem cells. Optimal sphere formation was observed when stem cells were cultured in the medium supplemented with EGF (20 ng/mL)/TGF*α* (20 ng/mL) [1, 25, 26]. In this study, we used the combination of EGF (20 ng/mL) + bFGF (20 ng/mL) + IGF-1 (50 ng/mL) to proliferate inner ear multipotent cells. Under this culture condition, the sphere cells displayed defining features of stem cells including high potential of proliferation and the capacity of self-renewal [27]. This capability of proliferation allowed the expansion of primary spheres through at least three passages. After the first passage, secondary spheres were formed from cells derived from a single primary sphere at a frequency of ~2 spheres, and the tertiary spheres could be formed at a frequency of ~1.5 spheres. However, the tertiary spheres were not expandable through additional passages, indicating that few cells in the spheres maintained the sphere formation capability. This finding supports a previous report that the proliferative capacity of cochlear stem cells decreases by 100-fold during the second and third postnatal weeks* in vivo* [7].

We identified three types of spheres during sphere formation, classified as solid, transitional, and hollow spheres. Cells derived from cochlear epithelia initially give rise to small solid spheres. These solid spheres were converted into transitional spheres and finally into hollow spheres. Diensthuber et al. [10] found that only solid spheres and, to a lesser extent, transitional spheres appeared to harbor self-renewing stem cells. The hollow spheres lost stemness and reduced the ability to give rise to hair cell-like cells. Therefore, we used the solid spheres for differentiation.

### 4.2. Induction of Differentiation toward an Inner Ear Progenitor Cell Lineage and Hair Cells

To determine whether multipotent cells in the spheres have the ability to ultimately differentiate into hair cells and supporting cells, we induced these cells under an adherent culture condition that included FGF3 and FGF10 in the first step induction and DAPT in the second step induction.

In most vertebrates, the FGF signaling pathway plays an important role in the early inner ear fate determination during development. In mice, the FGF signaling pathway is required for induction of the otic placode, and the ligands involved in the otic placode induction have been identified as FGF3 and FGF10 [28, 29]. We anticipated that exposure to these factors would trigger the differentiation of the multipotent cells into progenitor cells committed to the inner ear cell lineage. DAPT is a Notch signaling inhibitor that prevents the release of the Notch intracellular domain [30]. Following terminal mitosis, some of the prosensory cells begin to express Atoh1. The Atoh1-positive cells will upregulate the expression of Notch ligands and develop into hair cells. Expression of a Notch ligand leads to activation of Notch signaling in adjacent cells. As a result, Hes1 and Hes5 are expressed in neighboring cells that ultimately develop into supporting cells. Hes1 and Hes5 antagonize the expression of Atoh1, which is crucial for the inhibition of hair cell differentiation. DAPT can prevent the release of the Notch intracellular domain. In previous studies, DAPT treatment results in the downregulation of the Notch effectors Hes1 and Hes5 [31], increased Atoh1 transcriptional activity, and dramatically increased numbers of Atoh1-expressing cells and hair cells [32]. Therefore, the addition of FGFs and DAPT in subsequent differentiation media is likely to facilitate the desired differentiation program for each stage. The differentiation efficiency of inner ear multipotent cells into hair cells under the condition without any factor is very low [6, 7]. Only 8.7 ± 3.8% [6] or 3.6 ± 1.4% [7] of differentiated cells are labeled with an antibody for Myosin VIIA [7]. In our study, about 25.2 ± 3.1% of cells differentiated on poly-L-lysine for 14 days expressed Myosin VIIA. Our induction method significantly improves the differentiation efficiency of inner ear multipotent cells into hair cells.

### 4.3. Differentiation of Functional Hair Cells

In this study, two substrates were used for differentiating inner ear progenitor cells into hair cells. When the inner ear progenitor cells were plated onto poly-L-lysine, we observed Atoh1-positive cells that coexpressed hair cell marker Myosin VIIA. However, the cells induced on poly-L-lysine did not adopt the typical hair cell morphology, and Espin was distributed uniformly in the microvilli-derived F-acting structures of hair-cell-like cells (Figure 3(c), green, right). When the hair bundle-like structures on these cells were analyzed using SEM, stereocilia were found to be arranged randomly on the surface of hair-cell-like cells. These hair cell-like cells could also not be permeated rapidly by FM1-43FX, indicating that these cells were not functional hair cells. Therefore, expression of Atoh1 and Myosin VIIA was not sufficient for the formation of the typical hair bundle structure. We speculated that the* in vitro* culture condition containing only poly-L-lysine as the culture substrate did not provide a suitable environment for the differentiation of functional hair cells. The hair bundle formation should require complex cues that may be lacking in the poly-L-lysine culture condition.

White et al. [33] found the purified supporting cells cocultured with periotic mesenchymal cells can transdifferentiate into hair cells. The mitotically inactivated chicken utricle stromal cells were also used as feeder cells for the differentiation of Lgr5^+^ cells and supporting cells into hair cells [34, 35]. These hair cells displayed specific hair cell markers and stereocilia-like structures and were mechanosensitive. These results implicated the important role of stromal cells in supporting the differentiation of functional hair cells. In this study, in order to generate functional hair cells from multipotent cells in culture, we developed a two-step-induction culture condition. Firstly, inner ear multipotent cells were induced into progenitor cells of inner ear cell lineage features on the poly-L-lysine substratum. The progenitor cells were subsequently cultured on the mitotically inactivated chicken utricle stromal cells for differentiation of hair cells. Under this culture condition, the progenitor cells differentiate into defined patches of cells that harbored Espin-positive cells with F-actin-filled protrusions. The Espin immunoreactivity showed an asymmetrical distribution of the protein toward one side of the hair cells. Using SEM, we found that the hair-cell-like cells had a hair bundle-like structure consisting of numerous stereocilia and a single kinocilium. Moreover, the hair bundles also displayed other features characteristics of a hair bundle, such as interciliary links, asymmetric stereocilia tips, and tapering at the base. The hair-cell-like cells also contained functional mechanosensory channels demonstrated by the rapid permeation of FM1-43FX and the electrophysiological properties. These data together suggested the formation of functional hair cells by a two-step-induction condition* in vitro*, the induction of the inner ear progenitor fate followed by coculture with utricle stromal cells. We inferred that the chicken utricle stromal cells secrete various factors and cues that activate multiple signaling pathways to promote progression of hair cell differentiation including the formation of hair bundles and mechanotransduction channels. Therefore, some key factors which may be important in the coculture procedure should be researched further.

Our study suggested that multipotent cells are present in the neonatal mouse cochlear epithelia, and the cochlear multipotent cells could generate functional hair cells* in vitro* given appropriate conditions. The observation that* in vitro* generated hair-cell-like cells had stereocilia bundles and displayed voltage-dependent currents supports the fact that the generation of hair cells from mammalian inner ear multipotent cells is a feasible and promising approach for the development of stem cell-based treatment strategies for hearing and balance disorders.
